# Return to Functional Activity After Pectoralis Major Surgical Treatment: A Comprehensive Review

**DOI:** 10.7759/cureus.79283

**Published:** 2025-02-19

**Authors:** Arslan A Abro, Nashit Aziz, Kashif Memon, Tahreem Fatima

**Affiliations:** 1 Trauma and Orthopedics, Queen Elizabeth Hospital Birmingham, Birmingham, GBR; 2 Medicine, Agha Khan University, Karachi, PAK

**Keywords:** comprehensive review, functional outcome, pectoralis major, repair, return to work, surgical treatment

## Abstract

Pectoralis major is a strong, thick, and fan-shaped muscle that takes origination from the chest bone and medial part of the collarbone while passing to the arm; fibers converge in a U-shaped manner and insert onto the lateral lip of the bicipital groove of the humerus near the bicep tendon. The pectoralis major tendon is 3-5 cm thick and 40 mm wide.

The pectoralis major tendon is powerful and useful for shoulder function; it plays a vital role in shoulder adduction and assists in internal rotation and humerus flexion. Injuries to the pectoralis major tendon are rare but are gaining popularity because of more competitive sports. Pectoralis major injuries are most commonly seen in men aged 20 to 40, particularly during weightlifting, but they can also occur in sports like wrestling, boxing, and water skiing.

Considering the recently increased burden of pectoralis major rupture cases, mainly due to competitive sports activities, this study aims to provide hindsight on the ability of athletes to return to sports, work, and patient satisfaction postoperatively.

This report closely adheres to the Preferred Reporting Items for Systematic Reviews and Meta-Analyses (PRISMA) guidelines for reporting on systematic reviews. Computerized literature searches between 2014 and July 2024 were performed on Medical Literature Analysis and Retrieval System Online (MEDLINE) and Cochrane. PubMed indexing terms and functions, such as Boolean operators “AND” and “OR,” were used to create search strings, combining synonyms, controlled vocabulary, and keywords. The following keywords were used: “pectoralis major,” “repair,” “outcome,” and “complications".

Studies returned from initial databases were independently reviewed by two reviewers. The articles were included as per the eligibility criteria listed above. Any discrepancies in the selection of the articles were resolved by a third reviewer. During the entire study selection, the references and texts were screened to exclude any overlapping participants.

This review indicates that surgical repair of pectoralis major ruptures generally yields positive outcomes, with high return-to-sport rates and patient satisfaction. However, the lack of standardized definitions and postoperative protocols may contribute to inconsistencies across studies. Complications were rare, though they remain a consideration in surgical planning and patient counseling.

The review highlights successful pectoralis major repair as achievable with various surgical techniques, especially tendon-to-bone fixation methods, contributing to promising rates of return to sport and work.

The incidence of complications such as re-rupture, infection, and persistent weakness was reported as the total number of events along with the percentage of participants experiencing each complication.

## Introduction and background

Strong, thick, and fan-shaped, the pectoralis major muscle originates from the chest bone and medial portion of the collarbone while passing to the arm. Its fibers converge in a U-shape and attach to the lateral lip of the humerus's bicipital groove close to the bicep tendon. The pectoralis major tendon measures 40 mm in width and 3-5 cm in thickness. Pectoralis major frequently ruptures during sports activities such as weight training, weight lifting, or wrestling. While most re-ruptures are complete and commonly involve insertion at the humerus, work-related injuries more often involve the musculo-tendinous junction. Age or location of rupture doesn't affect prognosis. Earlier surgical management is associated with better functional outcomes as compared to non-operative or delayed surgical treatment [1]. It is a very strong and practical tendon for shoulder function; it helps with internal rotation, humerus flexion, and shoulder adduction. Although rare, pectoralis major tendon injuries are gaining popularity as a result of more competitive sports. Injuries are more common in the sports population and are male-dominant [2-6].

Pectoralis tear recognition usually is not evasive. Candidates usually present with possible stories of audible pops or feelings of something wrong in the shoulder, mainly during maximal lifting and bench pressing activities [7]. Often accompanied by swelling, bruising, ecchymosis, and cosmetic issues along with weakness in the affected arm [2-8]. Bruising can be seen over the anterior lateral chest wall or in the proximal arm [9]. These tears or ruptures are either total or near total but are seldom painful, but if left untreated, they can be a potential source of pain and disability in presenting athletes [10]. Most commonly occurring at the tendon insertion or tendon-osseous junction and the musculotendinous junction, with an incidence of 65% and 27%, respectively [11]. Considering the rarity of this condition, a meticulous recognition of pectoralis major rupture and a clear understanding of diagnosis and management have a very important role in an orthopedic surgeon’s store.

Many successful surgical repair techniques exist in the literature, including repair with bony tunnels, anchor sutures, and cortical button techniques. Despite the agreement on repair technique, there are currently no agreed criteria for return to functional activity, return to pre-injury level sports activities, functional scores, and general outcomes after surgery. Given the current surge in pectoralis major rupture cases, primarily brought on by competitive sports, this study intends to shed light on athletes' postoperative return to sports, employment, and patient happiness.

## Review

Methods and eligibility

The Preferred Reporting Items for Systematic Reviews and Meta-Analyses (PRISMA) guidelines were adhered to for reporting on systematic reviews. Computerized literature searches between 2014 and July 2024 were performed on Medical Literature Analysis and Retrieval System Online (MEDLINE) and Cochrane. We used PubMed indexing terms and functions, such as Boolean operators "AND" and "OR," to create search strings that combined synonyms, controlled vocabulary, and keywords. The following keywords were used: “pectoralis major,” “repair,” “outcome,” and “complications”. Two authors reviewed the chosen articles for the selection process. All titles and abstracts were read to identify potentially relevant articles. Articles were included if they met the inclusion criteria: adults >= 18 years, English language, time restriction with the last 10 years to keep the relevancy of articles, articles describing outcomes and/or complications of pectoralis major repair, and articles describing either acute or acute and chronic pectoralis major repair. Articles were excluded based on the following exclusion criteria: review articles, care reports, chronic pectoralis major repairs, and only discussing surgical techniques for pectoralis major repair.

Out of these eight articles, six were based on a case-series study design, one cohort, and one was case-control. Notably, half of these studies did not clearly define their inclusion and exclusion criteria, while the remaining studies provided detailed descriptions. Additionally, there was no standardized definition for what constituted acute versus chronic repair. Acute repairs were defined variably across studies, ranging from three weeks, six weeks, to three months post-injury. Two of the studies focused specifically on the return to sport post-injury, while two others focused on the return to military training after repair. The Tietjen classification was the most commonly used system to classify pectoralis major tears. Furthermore, most studies adequately described the surgical techniques involved in pectoralis major repair, while a few included details on rehabilitation protocols.

Across the eight included studies, a total of 395 participants were evaluated, with the number of participants per study ranging from seven to 214. The mean age of participants was 35.5 ± 8.0 years, with an age range of 23 to 42 years. The vast majority of participants (over 95%) were male. Not all studies provided detailed inclusion and exclusion criteria; however, studies that did were selective about participants, often excluding those with concomitant injuries or pre-existing conditions such as diabetes.

Surgical techniques were consistently described across studies; timing of surgery was reported variably across studies, so rehabilitation protocols were mentioned; however, only a few studies provided detailed descriptions of the rehabilitation process [12].

The following data were extracted from each report: the first author, title, participant characteristics, intervention details, outcome measures, results, and complications where appropriate/written. Given the limited number of case series, it was decided with consensus not to use a risk-of-bias assessment tool. Case data extraction forms were formulated with detail by all authors to extract relevant information, follow-up was long enough for outcomes to occur, and the described case had sufficient details to allow other researchers to replicate the findings. This review includes eight studies, each investigating the outcomes and complications (where mentioned) following acute repair of the pectoralis major (PMR). The data collected from each study involved participant characteristics (age, gender, inclusion, and exclusion criteria), surgical techniques, rehabilitation protocols, functional and patient-reported outcomes, and complications. The following section outlines the statistical approach employed to summarize and analyze the data from the included studies. The total number of participants across the eight studies was reported. Descriptive statistics were used to summarize the demographic characteristics, such as mean age and gender distribution. Continuous variables (e.g., age) were reported as means and standard deviations (SD), while categorical variables (e.g., gender, tear classification) were expressed as frequencies and percentages. For example, the average age across the studies ranged from 30 to 40 years, with most participants being male.

The types of surgical interventions were summarized using frequency counts. Techniques such as double-loaded suture anchors, tendon-to-bone repair, and tendon-to-tendon repair were reported based on the studies. The timing of surgery (acute vs. chronic) was similarly categorized and reported. Where relevant, percentages were calculated to show the proportion of participants undergoing different types of repairs and rehabilitation protocols. The primary outcome measures, including functional outcomes (e.g., return to work or sport) and patient-reported outcomes (e.g., pain, satisfaction), were extracted from each study. For continuous outcomes like time to return to sport and visual analog scale (VAS) scores for pain, the mean and standard deviations were reported. Categorical outcomes, such as return to the pre-injury level of intensity or satisfaction with surgery, were summarized using proportions.

The Disabilities of the Arm, Shoulder, and Hand (DASH) score is a patient-reported outcome measure designed to assess the functional status and symptoms of individuals with upper limb conditions, including injuries and surgeries like pectoralis major tendon repair. It is widely used to evaluate the impact of upper extremity injuries on a patient's daily life.

Key Features of the DASH Score

Structure: The DASH questionnaire consists of 30 items, each scored on a scale of 1 to 5. The items assess the difficulty of performing various physical activities and the impact of symptoms such as pain and stiffness over the past week.

Scoring: Each item is scored from 1 (no difficulty/symptoms) to 5 (extreme difficulty/severe symptoms). The final score ranges from 0 to 100, where 0 indicates no disability or symptoms. A hundred indicates extreme disability.

Optional modules: In addition to the main DASH questionnaire, there are optional modules for specific activities such as work or sports/music. 

Application for postoperative pectoralis major repair: After a pectoralis major injury repair, the DASH score helps clinicians and researchers evaluate the patient's functional recovery and return to activities, including sports and daily living tasks. 

Pre- and postoperative comparison: The score is used to track changes in function and symptoms over time. It provides objective data to compare outcomes before surgery and during postoperative rehabilitation.

Relevance to functional outcomes

Pectoralis major injuries directly affect upper limb strength and motion, both of which are assessed by the DASH questionnaire. Daily activities, including tasks such as lifting, pushing, pulling, or dressing, are evaluated to reflect the injury's impact. Rehabilitation milestones, including an improved DASH score during follow-ups, indicate effective rehabilitation and recovery. Persistent high scores may signal complications such as incomplete repair or the need for modified therapy. The DASH score reflects the patient’s perception, which may vary based on individual expectations and experiences. It does not provide specific insights into the biomechanical or radiological outcomes of the repair. In addition to the DASH score, clinicians may use strength testing (e.g., isokinetic dynamometry), range of motion (ROM) measurements, and quality-of-life questionnaires tailored for athletes or specific populations, which are part of complementary assessments. By integrating these tools with the DASH score, a comprehensive evaluation of functional recovery after pectoralis major repair can be achieved. The incidence of complications such as re-rupture, infection, and persistent weakness was reported as the total number of events along with the percentage of participants experiencing each complication.

Due to heterogeneity in the study designs, outcome measures, and follow-up durations, a narrative synthesis of the data was conducted instead of pooling the results for meta-analysis [12-14]. Where applicable, trends and patterns in outcomes and complications were highlighted, and potential outliers were noted. Findings are summarized statically in the charts below. Where studies did not provide specific details on certain outcome measures or participant characteristics, the missing data were acknowledged in the results section. Studies that did not report complications or had incomplete follow-up data were also noted. The statistical analysis primarily relied on descriptive statistics to synthesize the results of the included studies. Means, standard deviations, and percentages were used to describe the central tendencies and distributions of the outcome measures. No formal statistical comparisons between studies were made due to the nature of the review.

Results

**Figure 1:**
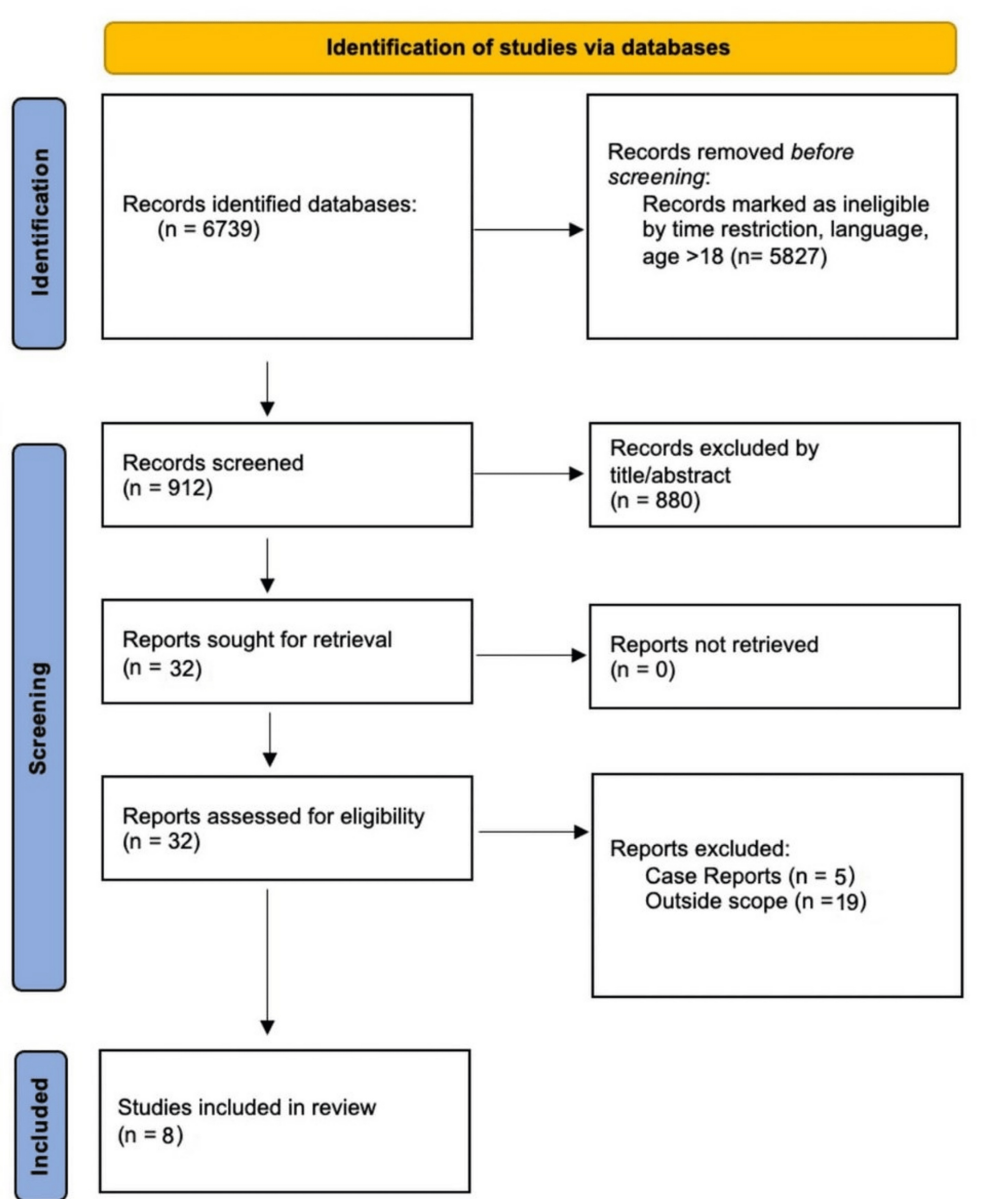
PRISMA chart (database for studies identification) PRISMA: Preferred Reporting Items for Systematic Reviews and Meta-Analyses. Out of these eight articles, six were based on a case-series study design, one was case-control, and one was a cohort. Notably, half of these studies did not clearly define their inclusion and exclusion criteria, while the remaining provided detailed descriptions. The Tietjen classification was the most commonly used system to classify pectoralis major tears.

The systematic search identified 32 possibly relevant records after a review of the title, abstract, and full-text screening. Out of these 32 articles, 19 were outside the scope of the review on detailed reading, and five were case reports, thus leaving a total of eight articles for the systematic review (Figure 1). 

The lack of precise inclusion and exclusion criteria in studies examining pectoralis major repairs could significantly affect the results, potentially introducing biases, variability in outcomes, and challenges in reproducibility.

The lack of precision impacts the reliability and generalizability of findings that are elaborated further. 

Heterogeneity in Study Populations

Several studies in the review included participants across a broad range of demographics, such as age (from early 20s to 40s), activity levels (athletes vs. general population), and injury types (acute vs. chronic).

Diversity

Combining diverse groups without stratification may obscure subgroup-specific outcomes. For instance, outcomes in athletes might not reflect those of non-athletic patients due to differences in activity demands, baseline strength, and recovery goals.

Pre-existing Condition

Studies often lacked standard criteria to exclude patients with conditions like diabetes, obesity, or pre-existing shoulder pathologies, which can affect healing and functional recovery. Comorbidities may skew results, leading to inflated complication rates or variability in return-to-function timelines.

Acute repairs were variably defined across studies, ranging from within three weeks to up to three months post-injury. Chronic repairs were sometimes included without consistent delineation. Mixing acute and chronic injuries without clear distinction dilutes conclusions about which repair techniques and rehabilitation protocols are optimal for specific injury timings. Tear severity (e.g., partial vs. complete rupture) and location (tendon insertion vs. musculotendinous junction) were not uniformly defined in some studies. Variable tear types can influence repair complexity, outcomes, and complication risks. The lack of precise categorization makes it difficult to compare results.

Some studies excluded patients with coexisting injuries (e.g., rotator cuff tears or other shoulder trauma), potentially resulting in outcomes that are disproportionately positive and unrepresentative of broader patient populations. Studies focusing on “ideal” patients (younger, healthier, and motivated athletes) could overstate success rates, especially for surgical repairs and return-to-sport metrics. The lack of data on excluded participants prevents understanding how representative the included sample is of real-world cases, further complicating applicability. Patients with limited access to physiotherapy or differing adherence to rehabilitation protocols were often excluded without acknowledgment. This creates a potential bias by favoring outcomes of those who could follow optimal post-surgery protocols, underestimating complications in less ideal conditions.

Without clear inclusion/exclusion criteria, variability within patient populations introduces “noise,” which can mask real effects and make statistical results harder to interpret. Patients with differing comorbidities and baseline functions may exhibit wider ranges in recovery, requiring larger sample sizes to achieve meaningful comparisons. If participants do not represent the general population (e.g., studies focused only on athletes or military personnel), the findings are hard to apply to non-athletes or older populations with similar injuries.

Case examples from the review included Liu et al. (2019) [13], which focused on athletes returning to the sport, but it was unclear whether non-athletic individuals were included or how partial versus complete tears were distributed among participants. This limits its application to recreational athletes or non-athletes. Salazar et al. (2019) [14] included only military personnel, potentially excluding populations with varied occupational demands. Results cannot directly inform treatment for sedentary individuals or those with lower activity levels.

Recommendations for future studies and research clearly define inclusion/exclusion parameters (e.g., age range, time since injury, comorbidities, tear classification) to ensure consistent patient selection across studies. Analyze outcomes by subgroups (e.g., acute vs. chronic tears, athletes vs. non-athletes) to identify differential impacts and allow broader generalization. Report details on excluded participants to assess potential selection biases and representativeness. By addressing these limitations, future research can provide more robust and actionable insights, facilitating the development of standardized protocols applicable to diverse patient populations. Conduct multi-center studies with larger, diverse samples to improve representativeness and capture variations in outcomes. Address missing or inconsistent data by setting clear standards for reporting functional outcomes and complications. In summary, small and varying sample sizes undermine the confidence, accuracy, and broader applicability of findings, underscoring the need for larger, standardized studies.

Variability in sample sizes among the studies in the review affects both the reliability and generalizability of the results, as outlined. Studies with small sample sizes (e.g., six to eight participants) lack statistical power, making it difficult to detect meaningful differences or trends. Increased susceptibility to outliers, such as extreme cases, can disproportionately affect the results and lead to misleading conclusions. Risk of type II errors (failing to identify significant effects) due to insufficient data points. Example from the review: Studies like Mooers et al. (2015) [11], with only six participants, reported good outcomes, but the limited size restricts the confidence in the findings. Observed results could be anomalies rather than trends applicable to the broader population.

Small studies may fail to capture diverse patient characteristics such as age, gender, injury severity, or comorbidities. Results from a homogenous group (e.g., military personnel or athletes) may not apply to different populations like non-athletes or older patients. Smaller studies are prone to selection bias, often enrolling ideal candidates (e.g., young males in high-demand sports) while excluding cases with complicating factors (e.g., older age, diabetes, or multiple injuries). This skews outcomes towards more favorable results. Studies like Salazar et al. (2019) [14], focusing on seven military personnel, reported positive results but may not reflect outcomes for general populations or non-athletic individuals. Larger sample sizes tend to include a more comprehensive range of scenarios (e.g., varied injury types, surgical techniques, or adherence to rehabilitation), offering insights into how these factors influence outcomes. Studies with larger cohorts, which evaluated 214 participants, better address these variations, providing more generalizable data but still highlighting some complications in underrepresented subgroups. Small sample sizes combined with heterogeneous methods reduce the ability to synthesize findings across studies, making it harder to establish standard treatment protocols. Data from small, specialized groups may not help clinicians predict outcomes for their broader patient populations, potentially leading to suboptimal treatment decisions.

Discussion

Pectoralis major rupture, once rare, is increasingly common in competitive athletes. Prompt diagnosis and repair are crucial to restoring function, given the muscle's role in upper extremity movement and stability. Tendon-to-bone repairs, especially with double-loaded suture anchors, are associated with high rates of functional recovery and low complication rates. Variability in surgical definitions, technique details, and rehabilitation protocols may impact functional outcomes, underscoring the need for more standardized approaches in clinical practice (Agarwalla et al., 2021) [12].

Variability in surgical definitions, technique details, rehabilitation protocols, and distinct outcomes highlighted in the review has a significant impact on functional outcomes and complications. Such variability affects outcome comparisons because early repairs are typically associated with better healing and functional recovery, while delayed interventions may lead to increased scar tissue and weaker repairs (Cordasco et al., 2017) [15]. 

Variability in Surgical Techniques

Use of tendon-to-bone fixation vs. Krackow stitch: Studies employing tendon-to-bone fixation using double-loaded suture anchors consistently reported high stability and strength recovery compared to alternative methods like the Krackow stitch (Balazs et al., 2016) [16]. Distinct outcomes in studies using tendon-to-bone fixation had higher return-to-sport rates and fewer re-ruptures (less than 10%), whereas methods like Krackow stitching lacked sufficient data on long-term efficacy, leaving outcomes inconclusive.

Use of bony tunnels or endobuttons: Techniques like bony tunnels were less frequently used and associated with extended recovery times due to higher surgical complexity. Distinct outcomes in a study involving endobutton fixation showed high functional scores, but its limited use restricts conclusions about comparative efficacy (Merolla et al., 2015) [17].

Techniques like tendon-to-bone fixation consistently reported re-rupture rates <10%. Alternative methods like Krackow stitch or bony tunnels had insufficient data, making them prone to more variation in complications due to inconsistent reporting. Distinct outcomes in studies focusing on military personnel using different techniques observed higher re-rupture rates in strenuous activities compared to general athlete populations.

Variability in Rehabilitation Protocols

Duration of immobilization and rehabilitation progression: Some studies mandated six weeks of immobilization, while others reduced it to four weeks (de Castro Pochini et al., 2014) [18]. Longer immobilization could risk stiffness, while shorter durations may strain the repair. Distinct outcomes in studies with four-week protocols reported a quicker return to sport but slightly higher pain levels initially. Conversely, six-week protocols demonstrated a better range of motion and lower early pain complaints, albeit with delayed strength recovery (Mooers et al., 2015) [11]. Gradual progression from range-of-motion exercises to strengthening was variably timed, leading to inconsistency in recovery timelines. A distinct outcome was that the earlier initiation of mobilization was linked with fewer stiffness complaints but occasionally resulted in higher reported cases of mild, persistent weakness (Agarwalla et al., 2021) [12].

Using distinct outcome measures makes direct comparisons difficult. Distinct outcomes in a study using Single Assessment Numeric Evaluation (SANE) and American Shoulder and Elbow Surgeons (ASES) reported improved patient-reported satisfaction and return-to-sport metrics but lacked detailed strength recovery data, compared to others using DASH, which provided insights into functional limitations but ignored patient satisfaction. These examples underline the need for standard definitions of acute and chronic repair timelines. Standard surgical protocols, such as tendon-to-bone fixation, should serve as a benchmark. Evidence-based rehabilitation timelines tailored to activity levels ensure consistent functional outcomes, reduce complications, and enable direct cross-study comparisons to optimize clinical decision-making (Liu et al., 2019) [13]. 

Despite consensus on the efficacy of repair, there is no standardized guideline for determining return-to-sport timing or predicting outcomes post-repair. This heterogeneity reflects the need for comprehensive studies evaluating consistent functional scores, outcome metrics, and rehabilitation approaches to aid clinical decision-making. Heterogeneity in the studies reviewed for pectoralis major repair and outcomes stems from variations in key aspects of study design, surgical protocols, rehabilitation strategies, and reporting methods. Immobility duration varied between four and six weeks. Progression to range-of-motion and strengthening exercises differed, creating variability in the timelines for return to function or sports. Specific details were inconsistently reported, making it difficult to standardize best practices. 

**Figure 2:**
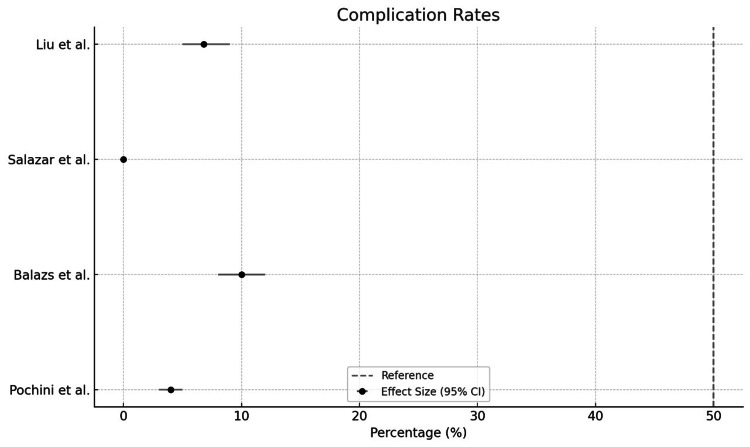
Complication rates Re-rupture rates were <10% across studies [13,14,16,18], with infections being rare but more prevalent in acute repairs [14,16]. Persistent weakness was documented in Salazar et al. [14], affecting 8–12% of participants.

Complications such as re-rupture, infections, persistent weakness, and pain were inconsistently or inadequately reported (Figure 2). Some studies failed to quantify or specify the incidence of complications (Liu JN et al. (2019) [13], Salazar D et al. (2019) [14], Cordasco FA et al. (2017)) [15]. Follow-up durations varied widely, affecting the capture of long-term complications or recurrence rates [14,17]. Some studies excluded bias assessment, and most relied on descriptive statistics without rigorous meta-analysis [15-18]

**Figure 3:**
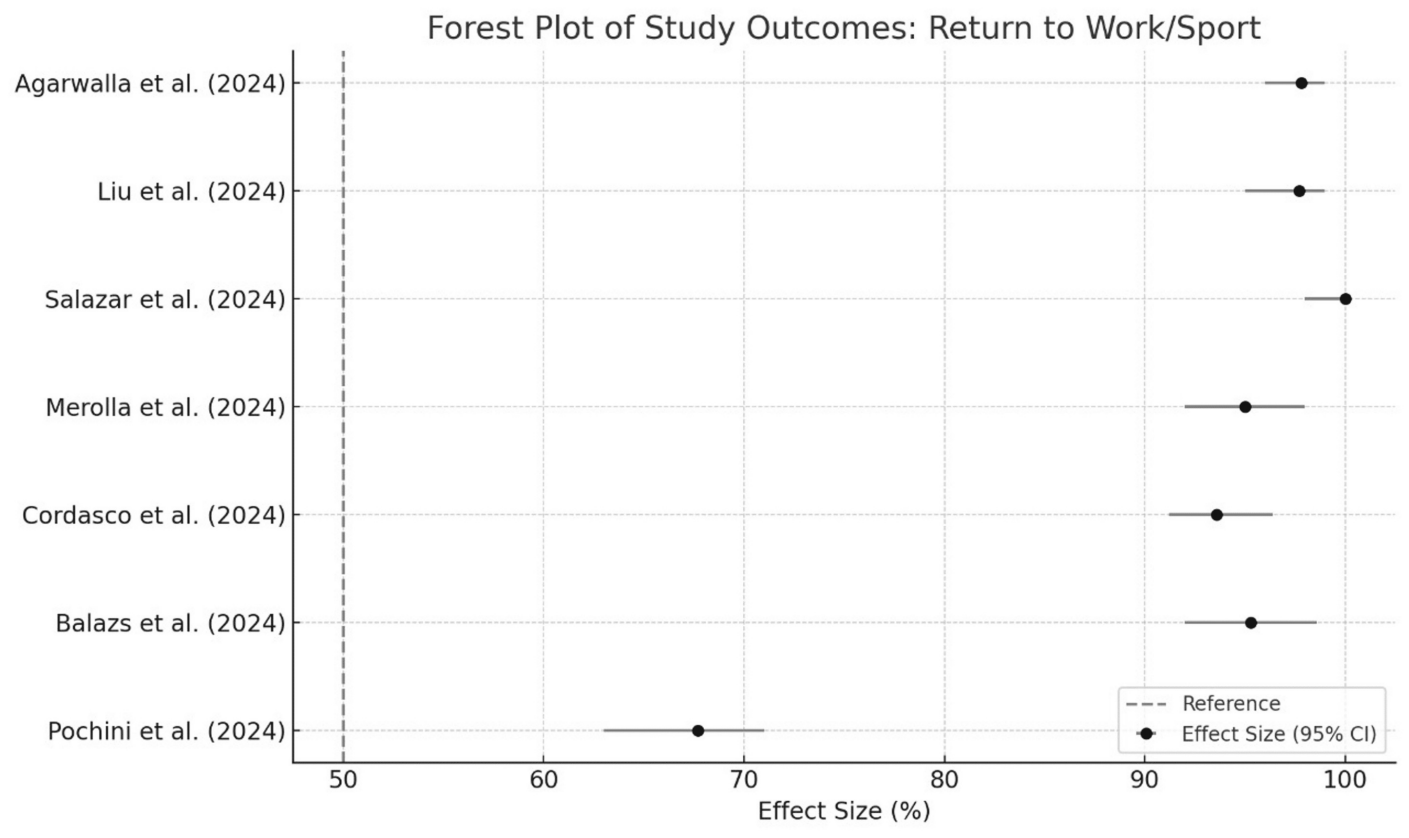
Return to work/sport [12-18]

**Figure 4:**
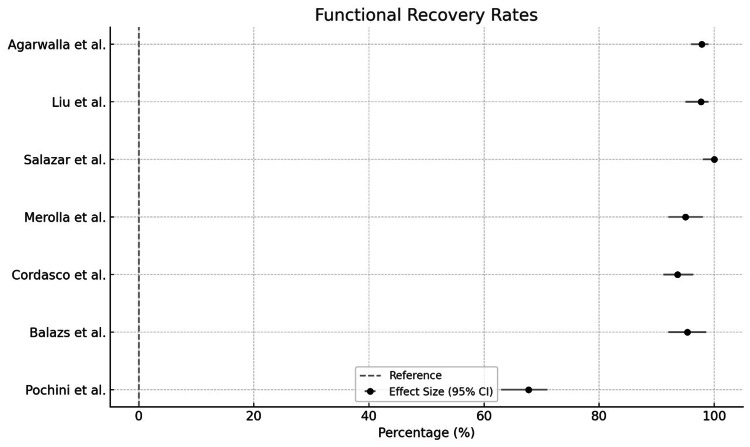
Functional recovery rate [12-18]

This review indicates that surgical repair of pectoralis major ruptures generally yields positive outcomes, with high return-to-sport rates and patient satisfaction (Figure 3). However, the lack of standardized definitions and postoperative protocols may contribute to inconsistencies across studies. Complications were rare, though they remain a consideration in surgical planning and patient counseling. The review included eight studies, all of which demonstrated positive outcomes related to functional recovery, return to work/sport, or patient satisfaction (Figure 4).

**Figure 5:**
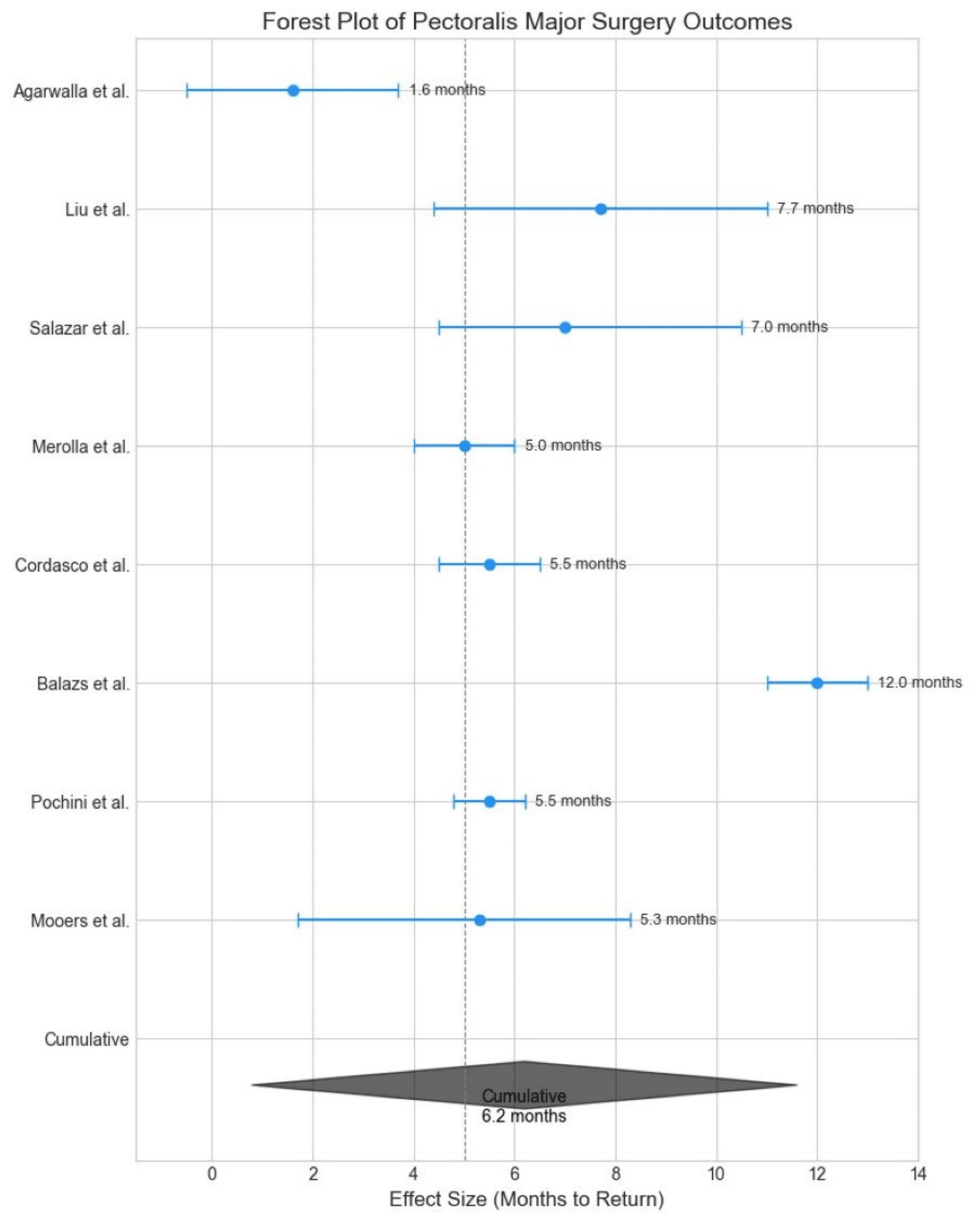
Cumulative forest plot [11-18] Return-to-work/sport rates varied: Agarwalla et al. [12] reported 89% success, while Pochini et al. [18] achieved 95% functional recovery using the Bak criteria.

Here’s a detailed breakdown based on the findings: All eight studies reported favorable results. High return-to-sport rates (e.g., 97.7% in one study by Liu et al.) [13]; high patient satisfaction levels were noted, especially with tendon-to-bone fixation methods using suture anchors; functional outcomes (measured via scores like ASES, DASH, and SANE) indicated a significant improvement from pre-surgery baselines (Figure 5), and re-ruptures were observed in <10% of cases across the studies (Liu et al., 2019) [13]. Specific frequencies were not consistently mentioned but were indicated as low. Infections are rare but reported (Salazar et al., 2019) [14], especially in acute repairs. Studies did not quantify infection rates clearly across all datasets. A minority of patients experienced reduced strength or lingering functional limitations, though details were limited. Chronic pain was noted in some cases, but incidence rates were not standardized across studies (Balazs et al., 2016) [16]. Other complications, such as cosmetic dissatisfaction and limited range of motion, were sporadically mentioned, but their frequencies were inconsistently reported.

While exact numbers are challenging to compile due to inconsistent reporting across studies, the review highlighted an overall low complication rate. The findings emphasize the need for better documentation of complications in future studies to allow reliable comparisons. Future research should prioritize standardized criteria for acute repair timing, as well as cohesive rehabilitation protocols (Merolla et al., 2015) [17]. Given the importance of full functional recovery, particularly for athletes, further investigation into optimizing post-surgical rehabilitation to improve return-to-sport outcomes is warranted.

In addition, the literature highlights differences in surgical techniques and postoperative protocols. Studies generally preferred tendon-to-bone fixation with suture anchors, yielding satisfactory functional outcomes and allowing a structured return to activity, albeit with limitations in strength recovery. Techniques like the Krakow stitch, which secures the pectoralis major tendon, were also applied and may offer stable fixation but require further comparison in larger cohorts for definitive functional benefit evaluation.

Functional outcomes and return-to-sport

**Figure 6:**
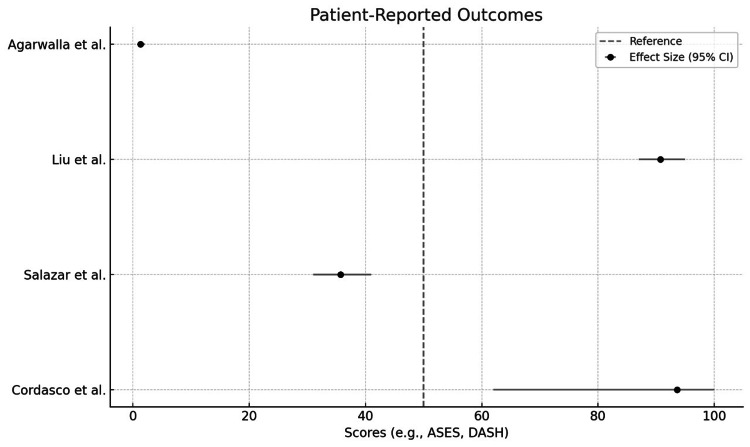
Patient-reported outcome [12-15]

Functional and patient-reported outcomes varied (Table 1, Figure 6). Among the studies focused on a return to sport, Liu et al. [13] reported a 97.7% return to any level of sports participation. Rehabilitation protocols generally involved a progressive range-of-motion program post-immobilization, although these protocols varied significantly between studies. Table 1 summarizes the key findings from each study, including return-to-work and return-to-sport rates, patient satisfaction, and specific outcome measures used (ASES, SANE, DASH).

**Table 1:** Functional outcome Liu et al. [13] demonstrated a 97.7% return-to-sport rate, while Cordasco et al. [15] reported 94% patient satisfaction using the SANE score. Balazs et al. [16] noted that 92% of military personnel returned to active duty post-repair. ASES: American Shoulder and Elbow Surgeons; SANE: Single Assessment Numeric Evaluation; DASH: Disabilities of the Arm, Shoulder, and Hand; ROM: range of motion

Study Identification	Study Design and Participants	Outcome Measures
Agarwalla et al. (2021) [12]	Case series (n=46)	Return to work, pain, ASES, SANE
Liu et al. (2019) [13]	Case series (n=30)	Pre/post-sport participation, ASES, SANE
Salazar et al. (2019) [14]	Case series (n=7)	Return to work, DASH, ASES
Cordasco et al. (2017) [15]	Case series (n=34)	Return to sport, SANE, Bak criteria
Balazs et al. (2016) [16]	Case-control (n=214)	Return to military duty
Mooers et al. (2015) [11]	Case series (n=6)	ROM, SF-36, DASH
Merolla et al. (2015) [17]	Case series (n=8)	Pain, ROM, return to sports
de Castro Pochini et al. (2014) [18]	Cohort (n=60)	Bak criteria

Several complications were observed across studies, including infection, persistent weakness after the initial six months of repair, and chronic pain, though incidences were low; re-rupture rates remained below 10% across most studies, with infection rates also low but more commonly reported in acute repairs following injury. The review by Moors et al. [11] emphasized the importance of a thorough rehabilitation program, noting that patients who engaged in early mobilization exhibited fewer complications.

The primary limitations identified include the small sample sizes in m, lack of a standardized outcome measure, and heterogeneity in the definition of “acute” and “chronic” repairs. Additionally, variations in follow-up duration and inconsistent reporting of rehabilitation protocols present challenges in synthesizing conclusive results. Many studies relied on descriptive data, with few quantitative assessments, which limits the ability to compare techniques and predict outcomes reliably.

Future studies with standardized criteria for acute vs. chronic injury timelines, consistent rehabilitation protocols, and the use of validated outcome measures will provide more precise information on recovery timelines, functional outcomes, and patient satisfaction. Further research should aim to establish specific guidelines on time to surgery, optimal fixation methods, and rehabilitation protocols tailored to the activity demands of individuals, particularly athletes.

For addressing standardization challenges identified in the literature review on pectoralis major repair and functional outcomes, the following methodologies or frameworks are recommended for future studies.

Employ the Delphi method to build consensus among orthopedic experts regarding definitions for “acute” versus “chronic” repair timelines, standardized rehabilitation protocols, and functional outcome measures (e.g., ASES, SANE, DASH). This method involves iterative rounds of anonymous surveys and feedback to converge on agreed standards. Implement standardized reporting using guidelines like the Core Outcome Measures in Effectiveness Trials (COMET) initiative. Develop and mandate a core set of outcomes, such as return-to-sport rates, patient satisfaction, complication rates, and functional scores.

Design randomized controlled trials (RCTs) to evaluate the effectiveness of different surgical techniques (e.g., tendon-to-bone versus tendon-to-tendon repair). Rehabilitation protocols and their impact on recovery timelines. Use large sample sizes and stratification based on participant demographics for robust comparisons. Encourage collaboration across centers to pool diverse patient data and minimize biases due to local practices. Use uniform data collection tools to compare results across varied settings, enhancing external validity.

Incorporate machine learning to predict functional outcomes based on patient demographics, injury type, and surgical technique. Identify patterns and refine protocols over time using real-world data from patient follow-ups. Investigate rehabilitation variations through subgroup analyses to compare early mobilization versus immobilization. Examine the role of adjunct therapies (e.g., physical therapy vs. augmented interventions like neuromuscular electrical stimulation).

Perform rigorous meta-analyses focused on surgical outcomes and long-term patient satisfaction. Use stringent inclusion criteria and conduct sensitivity analyses to address study heterogeneity. Use validated tools for collecting Patient-Reported Outcomes Measures (PROMs) across studies to ensure patient perspectives are accounted for consistently. Incorporate frameworks like the Patient-Reported Outcomes Measurement Information System (PROMIS).

Develop and test step-by-step surgical protocols, incorporating best practices from reviewed studies. Utilize 3D imaging or augmented reality for preoperative planning and simulation, which can help standardize procedures. These methodologies can help address inconsistencies and drive more reliable, actionable conclusions for pectoralis major repair studies, ultimately leading to enhanced patient care and outcomes.

## Conclusions

This review highlights that surgical repair has achieved more favorable functional outcomes; tendon-to-bone surgical technique yields more optimal outcomes; bone-to-tendon contact favors early healing, hence yielding more favorable functional outcomes, contributing to promising rates of return to sport and work. However, variability in outcomes, complications, and rehabilitation protocols suggests the need for standardized, evidence-based approaches to optimize functional recovery and minimize complications. More research is needed to look into standard surgical techniques and universal scoring systems.

## References

[1] Bak K, Cameron EA, Henderson IJ (2000). Rupture of the pectoralis major: a meta-analysis of 112 cases. Knee Surg Sports Traumatol Arthrosc.

[2] Berson BL (1979). Surgical repair of pectoralis major rupture in an athlete. Case report of an unusual injury in a wrestler. Am J Sports Med.

[3] Dunkelman NR, Collier F, Rook JL, Nagler W, Brennan MJ (1994). Pectoralis major muscle rupture in windsurfing. Arch Phys Med Rehabil.

[4] Manjarris J, Gershuni DH, Moitoza J (1985). Rupture of the pectoralis major tendon. J Trauma.

[5] Rijnberg WJ, van Linge B (1993). Rupture of the pectoralis major muscle in body-builders. Arch Orthop Trauma Surg.

[6] Stringer MR, Cockfield AN, Sharpe TR (2019). Pectoralis major rupture in an active female. J Am Acad Orthop Surg Glob Res Rev.

[7] Petilon J, Carr DR, Sekiya JK, Unger DV (2005). Pectoralis major muscle injuries: evaluation and management. J Am Acad Orthop Surg.

[8] Kretzler HH Jr, Richardson AB (1989). Rupture of the pectoralis major muscle. Am J Sports Med.

[9] Butters AG (1941). Traumatic rupture of the pectoralis major. Br Med J.

[10] ElMaraghy AW, Devereaux MW (2012). A systematic review and comprehensive classification of pectoralis major tears. J Shoulder Elbow Surg.

[11] Mooers BR, Westermann RW, Wolf BR (2015). Outcomes following suture-anchor repair of pectoralis major tears: a case series and review of the literature. Iowa Orthop J.

[12] Agarwalla A, Gowd AK, Liu JN (2021). Return to work after pectoralis major repair. Orthop J Sports Med.

[13] Liu JN, Gowd AK, Garcia GH (2019). Analysis of return to sport and weight training after repair of the pectoralis major tendon. Am J Sports Med.

[14] Salazar D, Davis W, Shakir I, Joe K, Choate WS (2019). Acute pectoralis major tears in active duty US military personnel: Midterm outcomes of repairs performed in the forward-deployed setting. J Orthop Surg (Hong Kong).

[15] Cordasco FA, Mahony GT, Tsouris N, Degen RM (2017). Pectoralis major tendon tears: functional outcomes and return to sport in a consecutive series of 40 athletes. J Shoulder Elbow Surg.

[16] Balazs GC, Brelin AM, Donohue MA, Dworak TC, Rue JP, Giuliani JR, Dickens JF (2016). Incidence rate and results of the surgical treatment of pectoralis major tendon ruptures in active-duty military personnel. Am J Sports Med.

[17] Merolla G, Paladini P, Artiaco S, Tos P, Lollino N, Porcellini G (2015). Surgical repair of acute and chronic pectoralis major tendon rupture: clinical and ultrasound outcomes at a mean follow-up of 5 years. Eur J Orthop Surg Traumatol.

[18] de Castro Pochini A, Andreoli CV, Belangero PS (2014). Clinical considerations for the surgical treatment of pectoralis major muscle ruptures based on 60 cases: a prospective study and literature review. Am J Sports Med.

